# Rare Titin (*TTN*) Variants in Diseases Associated with Sudden Cardiac Death

**DOI:** 10.3390/ijms161025773

**Published:** 2015-10-27

**Authors:** Oscar Campuzano, Olallo Sanchez-Molero, Irene Mademont-Soler, Helena Riuró, Catarina Allegue, Monica Coll, Alexandra Pérez-Serra, Jesus Mates, Ferran Picó, Anna Iglesias, Ramon Brugada

**Affiliations:** 1 Cardiovascular Genetics Center, University of Girona-IDIBGI, Girona 17190, Spain; E-Mails: oscar@brugada.org (O.C.); osanchezmolero@gencardio.com (O.S.-M.); imademont@gencardio.com (I.M.-S.); hriuro@gencardio.com (H.R.); callegue@gencardio.com (C.A.); mcoll@gencardio.com (M.C.); aperez@gencardio.com (A.P.-S.); jmates@gencardio.com (J.M.); ferran.pico@gencardio.com (F.P.); annai@brugada.org (A.I.); 2 Medical Science Department, School of Medicine, University of Girona, Girona 17071, Spain; 3 Cardiovascular Genetics Clinic, Hospital Josep Trueta, Girona 17007, Spain

**Keywords:** pediatric, juvenile, sudden cardiac death, genetics, next-generation sequencing, forensics, titin

## Abstract

A leading cause of death in western countries is sudden cardiac death, and can be associated with genetic disease. Next-generation sequencing has allowed thorough analysis of genes associated with this entity, including, most recently, titin. We aimed to identify potentially pathogenic genetic variants in titin. A total of 1126 samples were analyzed using a custom sequencing panel including major genes related to sudden cardiac death. Our cohort was divided into three groups: 432 cases from patients with cardiomyopathies, 130 cases from patients with channelopathies, and 564 post-mortem samples from individuals showing anatomical healthy hearts and non-conclusive causes of death after comprehensive autopsy. None of the patients included had definite pathogenic variants in the genes analyzed by our custom cardio-panel. Retrospective analysis comparing the in-house database and available public databases also was performed. We identified 554 rare variants in *titin*, 282 of which were novel. Seven were previously reported as pathogenic. Of these 554 variants, 493 were *missense* variants, 233 of which were novel. Of all variants identified, 399 were unique and 155 were identified at least twice. No definite pathogenic variants were identified in any of genes analyzed. We identified rare, mostly novel, titin variants that seem to play a potentially pathogenic role in sudden cardiac death. Additional studies should be performed to clarify the role of these variants in sudden cardiac death.

## 1. Introduction

Sudden cardiac death (SCD) is an unexpected death caused by a cardiac dysfunction. It is preceded by sudden loss of consciousness within one hour of the onset of acute symptoms [1]. The most common cause of SCD is myocardial infarction, usually in individuals 45–50 years of age or older [2]. In this wide age range, 5%–10% of SCD cases are due to inherited disorders, either cardiomyopathies (mainly hypertrophic cardiomyopathy (HCM), dilated cardiomyopathy (DCM), and arrhythmogenic right ventricular cardiomyopathy (ARVC)) or channelopathies (mainly long QT syndrome (LQT), Brugada syndrome (BrS), catecholaminergic polymorphic ventricular tachycardia (CPVT), and short QT syndrome (SQT)) [3].

Genetic analyses of SCD cases resulting from inherited diseases show that there are two main genetic associations: sarcomere/desmosome proteins, associated with cardiomyopathies; and ion channel or regulatory proteins, associated with channelopathies [4]. However, this simplistic classification is currently questioned because new genetic discoveries are more closely integrating sequencing data with diseases. For example, pathogenic variants in the *PKP2* gene have been reported in BrS patients [5,6], and pathogenic variants in the *SCN5A* gene have been reported in DCM cases [7].

**Figure 1:**
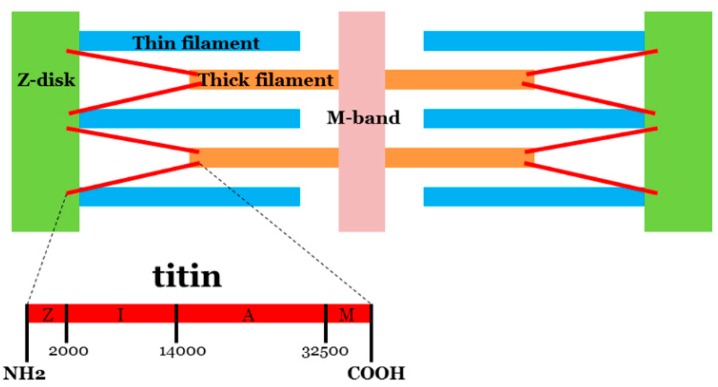
Representative diagram of titin’s position in myocyte architecture. Titin extends from the Z-disk of the sarcomere (N-terminus) to the M-band (C-terminus). The central part of the protein contains I-band region (I) and A-band region (A).

In heart (cardiac) muscle contraction, a main player is the protein titin (molecular mass of up to ~3800 kDa), encoded by the *TTN* gene (chromosome 2q31.2, ID: 7273, 363 exons). Titin extends from the Z-disk of the sarcomere (N-terminus) to the M-band (C-terminus) of the half sarcomere. The central part of the protein contains an elastic I-band region and a thick filament-binding A-band region (Figure 1). Although pathogenic variants in *TTN* have been associated mainly with DCM [8,9] and, to a lesser extent, other cardiomyopathies [10,11], and various skeletal myopathies [12], its large size hampered routine genetic analysis until the advent of next-generation sequencing (NGS) technology [13,14]. Recently, NGS studies in different cohorts have identified several variants of unknown significance (VUS) in *TTN*. The clinical role of these variants remains, for the most part, to be clarified [15].

In our study, we used NGS technology to analyze three cohorts of patients: a cohort of patients with cardiomyopathies, a cohort of patients with channelopathies, and a post-mortem cohort of individuals with structurally normal hearts after comprehensive autopsy. Our aim was to report all rare variants identified in the *TTN* gene and compare the prevalence of variants in these cohorts.

## 2. Results

Firstly, it is important to remark that all variants identified in our study were classified as rare following Minor Allele Frequency (MAF) minor 1% after consultation in population databases. Hence, all rare variants included in our study are not present in healthy population, including data of control samples available in public databases (no common variants in population—MAF > 1%—were analysed in our study). In addition, all these *TTN* rare variants are not identified in any of our other samples analyzed in our laboratory showing conclusive genetic cause of death.

**Figure 2:**
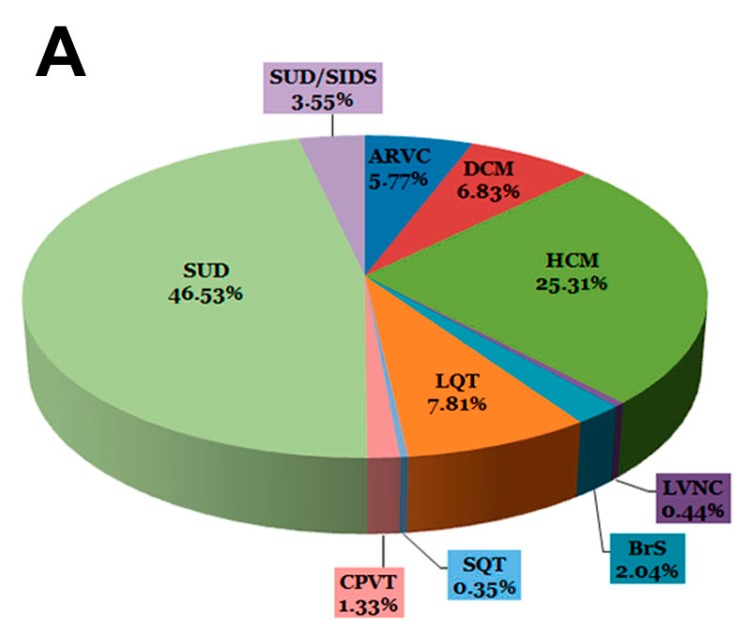

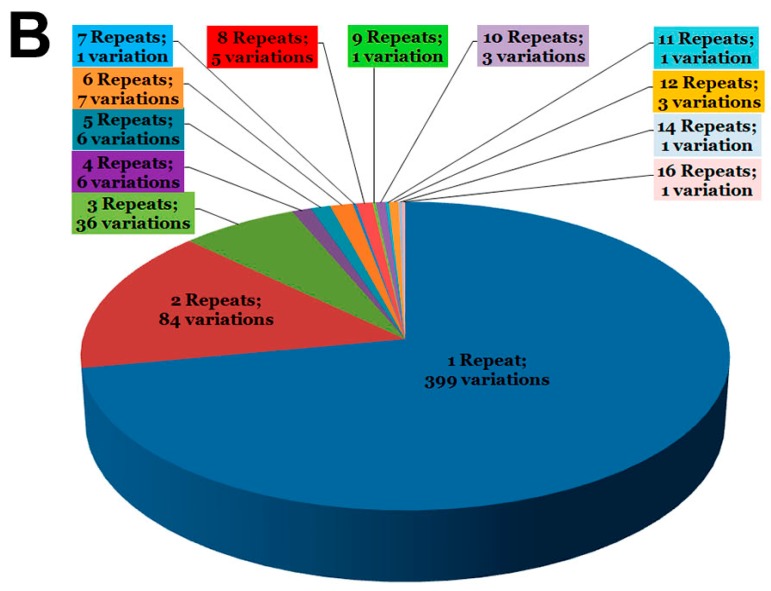
Representation of cases and variants. (**A**) Distribution of samples in the cohort; (**B**) Distribution of variants grouped by number of repetitions in the cohort. SUD: Sudden Unexplained Death; SIDS: Sudden Infant Death Syndrome; ARVC: Arrhythmogenic Right Ventricular Cardiomyopathy; DCM: Dilated Cardiomyopathy; HCM: Hypertrophic Cardiomyopathy; LQT: Long QT Syndrome; CPVT: Catecholaminergic Polymorphic Ventricular Tachycardia; SQT: Short QT Syndrome; BrS: Brugada Syndrome; LVNC: Left Ventricular Non-Compaction.

The cohort included 1126 samples divided into three groups: 130 patients with channelopathies (11.55%); 432 patients with cardiomyopathies (38.36%); and 564 individuals with sudden unexplained death (SUD) (50.09%) (Table 1; Figure 2A). The latter group was subdivided into individuals <one-year-old (SUD/SIDS (Sudden Infant Death Syndrome)) (40 cases; 3.55%) and all other cases (SUD) (524; 46.53%). In each sample analyzed, a median of four rare potentially pathogenic variants was identified in different genes analyzed using our custom-made panel. In all 1126 samples included in our study, no definite pathogenic variants were identified in any of the other genes analyzed using our custom-made panel.

Genetic analysis of the *TTN* gene identified 554 rare variants (18 intronic and 536 exonic), 282 of which were novel (8 intronic and 274 exonic) (Table 2 and Table S1). All variants were identified in heterozygosis. Regarding NGS data, a median of 830× was obtained. A minimum of 30× was considered for each variation (median call rate: 99.85). A median of eight failed exons occurred and all these exons were sequenced using Sanger technology.

**Table 1:** Samples included in our cohort of study. The SUDs group represents around 50% of all samples. Regarding live patients, around 25% correspond to HCM.

	Disease	Samples	Total
**Channelopathies**	BrS	23 (2.04%)	130 (11.55%)
	LQT	88 (7.81%)	
	SQT	4 (0.35%)	
	CPVT	15 (1.33%)	
**Cardiomyopathies**	ARVC	65 (5.77%)	432 (38.36%)
	HCM	285 (25.31%)	
	DCM	77 (6.83%)	
	LVNC	5 (0.44%)	
**SUDs**	SUD	524 (46.53%)	564 (50.09%)
	SUD/SIDS	40 (3.55%)	
		1126 (100%)	1126 (100%)

BrS, Brugada Syndrome; LQT, Long QT Syndrome; SQT, Short QT Syndrome; CPVT, Catecholaminergic Polymorphic Ventricular Tachycardia; ARVC, Arrhythmogenic Right Ventricular Cardiomyopathy; HCM, Hypertrophic Cardiomyopathy; DCM, Dilated Cardiomyopathy; LVNC, Left Ventricular Non-Compaction; SUD, Sudden Unexplained Death; SIDS, Sudden Infant Death Syndrome.

### 2.1. Intronic Variants

None of the 18 intronic variants were previously reported as pathogenic in the Human Gene Mutation Database (HGMD) or dbSNP. Two variants—c.32407+6C>T, rs72650067 and c.62012-5C>G, rs72646886—were identified three times in different diseases. Seven of the eight novel variants were identified only once in our cohorts (Figure 2B). One novel variant (c.50143+6T>A) was identified in three samples, all affected by HCM.

**Table 2:** Genetic variants in *TTN* identified in our cohorts. Most parts of variants were exonic and *missense*. Around 50% of all variants were novel. Curiously, regarding *nonsense* and *indels*, around 90% were novel.

Variants		
Intronic	18 (3.24%) Novel: 8 (1.44%)	-
Exonic	536 (96.75%) Novel: 274 (49.46%)	*Missense* 493 (88.99%) Novel: 233 (42.06%)
		*Nonsense* 6 (1.08%) Novel: 5 (0.90%)
		*Indels* 37 (6.68%) Novel: 36 (6.5%)
TOTAL	554 (100%) Novel: 282 (50.90%)	-

### 2.2. Exonic Variants

Of the 536 exonic variants, 274 were novel (51.12%), 493 were *missense* (91.98%) (233 novel; 43.47%), 37 were *indels* (6.9%) (36 novel; 6.72%), and 6 were *nonsense* (1.12%) (5 novel; 0.93%) (Table 2).

#### 2.2.1. Missense

All previously reported *missense* variants were considered VUS except six, which were previously classified as pathogenic (p.279R>W/rs138060032/myopathy with early respiratory failure; p.1034V>M/CM123527/muscular dystrophy; p.3751P>R/CM1310239/cardiac dysrhythmia; p.8848H>Y/CM116750/ARVC; p.18579A>T/CM116752/ARVC; and p.33291M>T/CM116755/ARVC). We identified these six *missense* variants only once except for p.3751P>R, which was identified six times in three different pathologies (Table S2), and p.279R>W, which was identified three times in two different pathologies (Figure 2B). Most previously reported *missense* variants were identified only once (138 of 260; 53.07%). In addition, 64 of 260 variants (24.61%) were identified twice, and, from these, only 28 of 64 *missense* variants identified twice occurred in the same pathology. All variants identified three or more times were identified in different pathologies (Figure 2B; Table S2).

Most novel *missense* variants (208 of 233; 89.27%) were identified only once. At least two of three *in silico* predictions classified all these novel variants as deleterious/damaging. Only five novel *missense* variants were identified twice in the same pathology (Figure 2B), and at least two of three *in silico* predictions classified all five as neutral/benign. All variants identified three or more times were identified in different pathologies, and at least two of three *in silico* predictions classified these novel variants as neutral/benign.

#### 2.2.2. Indels

We identified 37 *indels*. All were novel except one (c.100185_>A, rs281864930), which was previously reported as pathogenic (CD086152/tibial muscular dystrophy) but identified in our database in two different diseases (DCM and SUD) (Figure 2B). *In silico* prediction classified all novel *indels* as deleterious. All *indels* were identified only once in our database except eight variants, which were identified twice or more (Figure 2B). Of these eight variants identified more than once, three of them (c.97_>C, c.42106_>T, and c.57978_>C) were present in SUD samples (Figure 2B).

#### 2.2.3. Nonsense

Finally, we identified six *nonsense* variants. All six *nonsense* variants were identified only once in our database.

### 2.3. Cohorts

Dividing variants according to cohort (channelopathies, cardiomyopathies, and SUD) identified 99 variants in the 130 channelopathy samples (76.15%) (Table 3), 325 variants in the 432 cardiomyopathy samples (75.23%), and 296 variants (53.48%) in the 564 SUD samples. Some of these variants were repeated twice or more in different cohorts, so we excluded them from a possible association with, or modulation of, a specific pathology. We considered that only those rare *TTN* variants identified once or repeated in the same pathology could be associated, at least as a genetic modulator, with the risk of being affected. Therefore, in the SUD cohort, we identified 168 variants once and 24 variants twice in SUD and/or SIDS samples. In the channelopathy cohort, we identified 20 variants once in BrS samples, 29 variants in LQT samples, seven variants in CPVT samples, and two variants in SQT samples. In the cardiomyopathy cohort, we identified 113 variants once and six variants twice in HCM samples. In DCM samples, we identified 32 variants once and three variants twice. In ARVC samples, we identified 26 variants once and two variants twice. Finally, two variants were identified only once in LVNC samples (Table 3).

## 3. Discussion

NGS technology facilitates the discovery of novel genetic alterations in a rapid and cost-effective way but translation into clinical practice should be performed carefully [16]. Currently, the main challenge is to discern pathogenic variants from background variants [17]. Although genetic analysis of *TTN* has been scarce so far, recent disease associations have launched its study in clinical scenarios. Most reports have focused on radical (*nonsense* and *indels*) *TTN* variants [8,18], ignoring *missense* variants despite the fact that they encompass most identified variants. Our study reports rare variants in *TTN* after NGS analysis of main genes associated with SCD. We performed analyses to assess the prevalence of variants in three different cohorts to help discern whether each variant has potential clinical significance, at least as a disease modulator [19]. In addition, although *TTN* has been mainly associated with cardiomyopathies [20,21], we included channelopathies in the analyses due to recent potential associations between structural proteins and ion channel diseases, such as *PKP2* and BrS [5,6], and in concordance with identification of potentially pathogenic structural gene variants in SIDS cases [22]. Due to *TTN*’s large size, variants could induce alterations in ion channels because the protein has several interactions with other cellular proteins [23]. Taking all these data into account, we believe that this large cohort enables a consistent and comprehensive study of *TTN* variants and helps clarify, regarding frequency data, the potentially pathogenic or benign role of these variants in SCD diseases.

**Table 3:** Relation of total of samples and total of variants identified only one time in the *TTN* gene. Inside the table there is the number of variants in *TTN* only identified one time, and in each disease.

			Total Samples 1126								
			Channelopathies 130				Cardiomyopathies 432				Post-Mortem 564
			BrS 23	LQT 88	SQT 4	CPVT 15	HCM 285	DCM 77	ARVC 65	LVNC 5	
**Total Variants 554**	Post-Mortem 296	-	-	-	-	-	-	-	-	-	168 (56.75%)
	Cardiomyopathies 325	LVNC 5	-	-	-	-	-	-	-	2 (40%)	-
		ARVC 53	-	-	-	-	-	-	26 (49.05%)	-	-
		DCM 64	-	-	-	-	-	32 (50%)	-	-	-
		HCM 203	-	-	-	-	113 (55.65%)	-	-	-	-
	Channelopathies 99	CPVT 15	-	-	-	7 (46.66%)	-	-	-	-	-
		SQT 4	-	-	-	-	-	-	-	-	-
		LQT 57	-	29 (50.87%)	-	-	-	-	-	-	-
		BrS 23	20 (86.95%)	-	-	-	-	-	-	-	-

We identified 554 rare *TTN* variants, mostly *missense* variants, in 1126 independent samples. As mentioned above, we use MAF minor 1% to classify a variant as rare (not common), following international guidelines/recommendations. Despite this fact, our group believes that the MAF threshold to discern rare and common variants should be, at least, 0.5%. For this reason, and in order to be less permissive, we excluded variants repeated at least three times in three different pathologies. The remaining variants, identified only once or repeated twice in different samples with the same disease, could therefore potentially be causative of disease or be a phenotypic modifier. Curiously, approximately 50% of all identified variants in each disease were repeated only once, except in patients suffering with BrS. In patients diagnosed with this channelopathy, 86.95% of identified variants were only repeated once, suggesting a potential role of *TTN* variants in BrS, at least as a genetic modifier, despite further studies should be performed to clarify this point. This relationship could be supported by the recent association of structural myocyte proteins with BrS [5,6]. However and as previously mentioned, future genotype–phenotype correlation studies in relatives will be crucial to elucidate the role of *TTN* variants in SCD. In addition, to clarify their pathogenic role, Giudicessi *et al.* [24] proposed the use of at least three *in silico* tools for pathogenic prediction of rare variants. In general, most VUS repeated three or more times showed diverse *in silico* predictions, while VUS identified only once or twice were predicted consistently as pathogenic/deleterious. However, all predictions obtained *in silico* should be translated into clinical practice with caution. *In silico* prediction is based on mathematical algorithms, and a recent report from our group showed that there is not always concordance between bioinformatic prediction and physiological role and that predictions can produce erroneous conclusions regarding pathogenicity [25]. In addition, both *nonsense* and *indel* variants are considered potentially damaging/deleterious *per se*, although most reported variants do not have corresponding mechanistic studies to show pathogenic physiological effects [8,26]. Regarding intronic variants, so far only a small number of variants have been classified as pathogenic. Most remain classified as VUS due to a lack of mechanistic studies to elucidate their role in clinical phenotypes. A recent study of Roberts *et al*. conclude that *nonsense*, frameshift, and canonical splice-site *TTN* variants inducing a truncated protein can be considered pathogenic; however, truncations that occur in novel-specific exons or other infrequently used *TTN* exons are less likely to be deleterious [26]. Despite this improvement in genetic classification, further studies should be performed to confirm this classification. Consequently, before clinical interpretation, we believe that a combination of several studies—including familial segregation, *in vitro* and *in vivo* analyses, and genetic variant frequency analyses in global populations—should be performed to conclude a definite pathogenic role.

### 3.1. Clinical Implications

Currently, genetic analysis of *TTN* for diagnosed or suspected cases involving cardiomyopathies should be performed because it is the main gene responsible for DCM. Despite this fact, only radical variants have been definitively classified as pathogenic. Hence, genetic data obtained after *TTN* analysis is of limited clinical value to adopt preventive and therapeutic strategies in rare *missense* variants without family segregation. Regarding clinically diagnosed channelopathies or SCD cases with no heart alterations identified during autopsy, variants in *TTN* should be considered as incidental findings not directly associated with any channelopathy. It is essential that a cautious genetic analysis that considers clinical data, situation surrounding death, and family history is made in order to try to elucidate the role of *TTN* variants in channelopathies and SCD cases. The American College of Medical Genetics and Genomics (ACMG) has suggested that such ambiguous genetic data unrelated to the diagnostic evaluation should not be returned to families [27]. However, the lack of currently elucidated mechanistic pathways suggests that *TTN* variants could modulate phenotype, and our results suggest that *TTN* variants may indeed play a role in numerous pathologies.

### 3.2. Limitations

This study’s main limitation is family segregation. Genetic and clinical assessment of family members should be performed to elucidate, at least in part, the role of VUS identified here, in concordance with the suggested necessity of cosegregation and functional data in interpretation of VUS [28]. Other limitations are the lack of *in vitro/in vivo* studies of identified VUS. Without results of both analyses, a low probability of pathogenicity should be considered in all VUS, and clinical translation should be implemented with caution. Finally, despite elevated number of genes analyzed, in our custom panel there are still many missing genes associated with some of the phenotypes included.

## 4. Experimental Section

### 4.1. Cohort

Our laboratory analyzed more than 2000 European samples (clinically diagnosed with any channelopathy, cardiomyopathy or sudden death associated diseases, even post-mortem cases diagnosed unexplained after comprehensive post-mortem autopsy) using a new NGS custom panel (Table 1). The present cohort includes a total of 1126 European samples (no familial relation between any of them) in which no rare causal genetic variant was identified (Table S1).

One segment of the cohort included 562 live patients clinically diagnosed with different arrhythmogenic pathologies before genetic analysis: 130 of which were associated with channelopathies (BrS, LQT, SQT, and CPVT) and 432 of which were associated with cardiomyopathies (HCM, DCM, LVNC, and ARVC) (Table 1). Another segment of the cohort included 564 post-mortem blood samples of sudden unexplained death (SUD), which had a non-conclusive cause of decease after complete autopsy, including 40 cases of sudden infant death syndrome (SUD/SIDS). Toxicological analyses were negative and macroscopic and microscopic analysis did not identify any heart alteration that could be the cause of death. This study was approved by the Ethics Committee of Hospital Josep Trueta (Girona, Spain) and conforms to the principles outlined in the Declaration of Helsinki. Alive individuals signed a written informed consent to participate in the study. Informed consent of all patients was obtained in accordance with international review board guidelines of Hospital Josep Trueta and Universitat of Girona (Girona, Spain). Regarding the post-mortem cohort, all samples followed a juridical process and legal consent of forensic was obtained only for research purposes. All samples were anonymized.

### 4.2. Samples

Genomic DNA was extracted with the Chemagic Magnetic Separation Module I (PerkinElmer Inc., Waltham, MA, USA) from post-mortem or fresh whole blood. Spectrophotometric measurements were performed to assess DNA quality using NanoDrop^®^ ND-1000 UV-Vis (Thermo Fisher Scientific, Waltham, MA, USA). Only DNA samples with 260/280 and 260/230 absorbance ratios of ≥1.8 were included. DNA concentration was determined by fluorometry with Qubit (Life Technologies, Waltham, MA, USA), and DNA integrity was assessed on a 0.8% agarose gel.

DNA was fragmented with a Bioruptor (Diagenode, Denville, NJ, USA). Library preparation was performed with SureSelect XT Custom 0.5–2.9 Mb library according to manufacturer’s instructions (Agilent Technologies Inc., Santa Clara, CA, USA). After capture, indexed libraries were sequenced in six-sample pools per cartridge. Sequencing paired-end process was developed on MiSeq System (Illumina Inc., San Diego, CA, USA) using a read length of 2 × 76 bp.

### 4.3. Custom Sequencing Panel

We used a custom-made panel of genes associated with SCD (*ACTC1*, *ACTN2*, *ANK2*, *CACNA1C*, *CACNB2*, *CASQ2*, *CAV3*, *CRYAB*, *CSRP3*, *DES*, *DMD*, *DSC2*, *DSG2*, *DSP*, *EMD*, *FBN1*, *GLA*, *GPD1L*, *HCN4*, *JPH2*, *JUP*, *KCNE1*, *KCNE2*, *KCNH2*, *KCNJ2*, *KCNQ1*, *LAMP2*, *LDB3*, *LMNA*, *MYBPC3*, *MYH6*, *MYH7*, *MYL2*, *MYL3*, *MYOZ2*, *PDLIM3*, *PKP2*, *PLN*, *PRKAG2*, *RYR2*, *SCN4B*, *SCN5A*, *SGCA*, *SGCB*, *SGCD*, *TAZ*, *TCAP*, *TGFB3*, *TGFBR2*, *TNNC1*, *TNNI3*, *TNNT2*, *TPM1*, *TTN*, and *VCL*), as previously reported by our group [29]. Genomic coordinates were designed using eArray (Agilent Technologies). All gene isoforms described in the University of California, Santa Cruz (UCSC) browser were included in the design. Biotinylated cRNA probe solution was used as a capture probe (Agilent Technologies). The final size was 432,512 kb of encoding regions and UTR boundaries. Coordinates of sequence data were based on NCBI build 37 (UCSC hg19). This custom resequencing panel is commercialized by Ferrer inCode as SudD inCode^®^.

### 4.4. Bioinformatics

Bioinformatic analysis of the data included a first step trimming of the FAST-Q files with an in-house developed method. Processed reads were mapped with GEM III (Available online: http://algorithms.cnag.cat/wiki/The_GEM_library), and the output from mapping steps was joined and sorted. Only uniquely and properly mapped read pairs were selected. Finally, the cleaned BAM file was annotated with SAMtools v.1.18 (Available online: http://samtools.sourceforge.net/) and GATK v3.2/2 (Available online: https://www.broadinstitute.org/gatk/index.php), together with a method developed in-house to generate the first raw variant call format (VCF) files. Variants were annotated with dbSNP IDs, Exome Variant Server (EVS), The 1000 Genomes Browser, and Ensembl information, if available. Recent published database ExAC (Available online: http://exac.broadinstitute.org/) was also consulted but, so far no data was available regarding *TTN*. In addition, in-house database including more than 2000 European samples was consulted for allele frequency of each variant. In addition, allelic frequency for each genetic variation identified was consulted in EVS, and 1000 Genomes database. The Human Gene Mutation Database (HGMD) (Available online: http://www.hgmd.cf.ac.uk/ac/index.php) was consulted to identify previously reported pathogenic mutations. *In silico* prediction of pathogenicity of novel genetic variants was assessed using Protein Variation Effect Analyzer (PROVEAN) (Available online: http://provean.jcvi.org/index.php), Polymorphism Phenotyping V2 (PolyPhen-2) (Available online: http://genetics.bwh.harvard.edu/pph2/), and Mutation Taster (Available online: http://www. mutationtaster.org/). Alignment of DNA sequences for different species also was performed for novel variants using Uniprot database (Available online: http://www.uniprot.org/).

### 4.5. Sanger Sequencing

Confirmation of non-common genetic variants (minor allele frequency, MAF < 1%) identified in NGS analysis was performed using Sanger sequencing. The genetic study included direct sequencing in both directions using the Big Dye Terminator v3.1 cycle sequencing kit and 3130XL Genetic Analyzer of exons and exon–intron boundaries (±10 nucleotides) amplified by Verities PCR (Applied Biosystems, Austin, TX, USA). We used NM_133378.4 (CCDS 54424.1) as the reference sequence for *TTN* and compared obtained data with the reference sequence from hg19 with Posterior SeqScape Software V2.5 (Life Technologies). The identified variants were compared with HGMD (Available online: http://www.hgmd.cf.ac.uk/ac/index.php), HapMap (Available online: http://hapmap.ncbi.nlm.nih.gov/), The 1000 Genomes (Available online: http://www.1000genomes.org/), and EVS (Available online: http://evs.gs.washington.edu/EVS/). Sequence variants were described following HGVS rules (Available online: http://www.hgvs.org/) and checked in Mutalyzer (Available online: https://mutalyzer.nl/).

## 5. Conclusions

We have identified a large number of VUS in the *TTN* gene from a cohort of samples from patients suffering cardiac diseases associated with SCD or suddenly dead with suspicions of cardiac arrhythmia. A high proportion of these VUS was identified only once or repeated in the same pathology, suggesting a potentially pathogenic or genetic modulator role. However, lack of definitively pathogenic interpretation implies careful clinical translation. Comprehensive genotype–phenotype studies in families suffering with cardiac disease are necessary to clarify the clinical role of these VUS in the *TTN* gene.

## Supplementary Materials

Table S1: All rare variants identified in *TTN* from genetic analysis of the cohort; Table S2: *In silico* data of *TTN* variants with four or more repeats from genetic analysis of the cohort. Supplementary materials can be found at http://www.mdpi.com/1422-0067/16/10/25773/s1.
