# Supplementary material for: Rare Titin (TTN) Variants in Diseases Associated with Sudden Cardiac Death
Source: Int J Mol Sci. 2015 Oct 27;16(10):25773–87. doi: 10.3390/ijms161025773 (PMC4632826; doi:10.3390/ijms161025773)
Supplement: Supplementary file 1 [file ijms-16-25773-s001.zip › ijms-94850-Supplementary Information/Table S2.pdf]

Supplementary Information

Table S2. Titin Variants included in our cohort of study showing 4 or more repeats. Data reported and *in silico* prediction do not allowed a conclusive role.

Protein Change	Disease	Repeats	Different Disease	dbSNP/ClinVar	HGMD	MAF (%) (EA/AA/All)	Polyphen-2	Mutation Taster	Provean
p.1345G>D	ARVC, DCM, SUD (2), HCM	5	yes	rs36021856/Likely benign	NA	0.7326/0.0681/0.5075	Probably Damaging	Disease Causing	Deleterious
p.1441R>P	LQT, SUD (4), DCM	6	yes	rs72647876/Benign	NA	0.7558/0.227/0.5767	Probably Damaging	Disease Causing	Deleterious
p.2917T>P	HCM (6)	6	no	Novel	NA	NA	Probably Damaging	Polymorphism	Deleterious
p.3405A>T	ARVC, HCM (7)	8	yes	rs6433728	NA	0.0/0.2634/0.0831	Benign	Disease Causing	Neutral
p.3713T>S	ARVC (2), HCM, SQT, DCM, SUD (7)	12	yes	rs72648925/Likely benign	NA	0.3536/0.0541/0.2605	Benign	Polymorphism	Neutral
p.3751P>R	SUD (2), SUD/SIDS, ARVC, HCM (2)	6	yes	rs72648927/Likely benign	CM1310239 /Cardiac dysrhythmia	0.9016/0.1613/0.6707	Probably Damaging	Polymorphism	Deleterious
p.4191V>M	SUD (2), SUD/SIDS, ARVC, HCM (2)	6	yes	rs72648937/Likely benign	NA	0.9062/0.6368/0.8195	Benign	Polymorphism	Neutral
p.5771A>V	ARVC, DCM, SUD (3), HCM	6	yes	rs72648960/Benign	NA	0.3149/0.1567/0.2648	Benign	Polymorphism	Neutral
p.6301R>Q	HCM (2), SUD (2)	4	yes	rs72648969/Likely benign	NA	0.4/0.07/0.2	Probably Damaging	Polymorphism	Neutral
p.6500N>K	ARVC (2), DCM, SUD (2)	5	yes	rs72648972/Likely benign	NA	0.2089/0.0274/0.1527	Benign	Polymorphism	Neutral
p.7599T>M	ARVC, SUD (3), HCM	5	yes	rs72648990/ Uncertain significance	NA	0.0856/0.0/0.0591	Benign	Polymorphism	Deleterious
p.9631P>L	ARVC, SUD (8), SUD/SIDS, CPVT, DCM, HCM (2)	14	yes	rs72650031/ Uncertain significance	NA	0.5875/0.0818/0.4308	Benign	Disease Causing	Deleterious
p.9895R>T	SUD (4), HCM (4)	8	yes	rs72650040/Likely benign	NA	0.3282/0.0267/0.234	Benign	Polymorphism	Neutral
p.10161V>L	DCM, HCM (2), LQT (3), SUD (4)	10	yes	Novel	NA	NA	Benign	Polymorphism	Neutral

Table S2. *Cont.*

Protein Change	Disease	Repeats	Different Disease	dbSNP/ClinVar	HGMD	MAF (%) (EA/AA/All)	Polyphen-2	Mutation Taster	Provean
p.10801P>R	ARVC, DCM, SUD (3), HCM	6	yes	rs72650066/Benign	NA	0.3414/0.1058/0.267	Benign	Polymorphism	Neutral
p.11967R>C	SUD (3), HCM, LQT	5	yes	rs12471771/Benign	NA	0.0722/0.2063/0.1149	Probably Damaging	Disease Causing	Deleterious
p.11996S>T	SUD, ARVC, HCM, SUD/SIDS	4	yes	rs181189778/ Uncertain significance	NA	0.0/0.0805/0.0251	Probably Damaging	Disease Causing	Neutral
p.13163R>C	ARVC, SUD (2), HCM	4	yes	rs72677231/ Uncertain significance	NA	0.2889/0.0517/0.2136	Probably Damaging	Disease Causing	Deleterious
p.13340R>H	HCM (6), DCM, SUD (4), SUD/SIDS	12	yes	rs72677237	NA	0.8798/0.1616/0.6556	Probably Damaging	Disease Causing	Deleterious
p.20476P>S	ARVC (2), DCM, LQT (2), SUD, HCM (4)	10	yes	rs55980498/Likely benign	NA	0.1/0.5/0.4	Probably Damaging	Disease Causing	Deleterious
p.20658S>G	HCM (6), DCM, SUD (4), SUD/SIDS	12	yes	rs72646885	NA	0.9183/0.1033/0.6585	Probably Damaging	Disease Causing	Deleterious
p.21743T>A	SUD, HCM, SUD/SIDS (2)	4	yes	rs56201325/ Uncertain significance	NA	0.5194/0.0523/0.3718	Benign	Polymorphism	Deleterious
p.22041E>Q	CPVT, HCM (3), SUD (2)	6	yes	rs55762754/ Uncertain significance	NA	0.2413/0.0259/0.173	Probably Damaging	Disease Causing	Neutral
p.25204N>I	LQT, LVNC, SUD, HCM (5)	8	yes	Novel	NA	NA	Probably Damaging	Disease Causing	Deleterious
p.26315E>K	SUD (8)	8	no	Novel	NA	NA	Probably Damaging	Disease Causing	Deleterious
p.26994G>D	ARVC, SUD (3), HCM	5	yes	rs72648235	NA	0.0239/0.0/0.0162	Probably Damaging	Disease Causing	Deleterious
p.27228V>M	HCM (4)	4	no	rs72648237/ Uncertain significance	NA	0.0362/0.0/0.0247	Probably Damaging	Disease Causing	Neutral

Table S2. *Cont.*

Protein Change	Disease	Repeats	Different Disease	dbSNP/ClinVar	HGMD	MAF (%) (EA/AA/All)	Polyphen-2	Mutation Taster	Provean
p.27957I>V	DCM, SUD (2), ARVC, BrS, HCM (2), SUD/SIDS (2)	9	yes	rs72648244	NA	0.9688/0.2765/0.7562	Benign	Polymorphism	Neutral
p.28163I>V	SUD (4), SUD/SIDS (2), HCM (2), LQT, SUD	10	yes	rs16866391/Benign	NA	0.1572/0.285/0.1978	Benign	Polymorphism	Neutral
p.28999D>E	SUD, ARVC (2), HCM, SQT, SUD (5), DCM	11	yes	rs72648253	NA	0.3499/0.0773/0.263	Benign	Disease Causing	Neutral
p.30019R>H	ARVC (2), LQT, SUD (2), DCM, HCM	7	yes	rs55704830/ Uncertain significance	NA	0.3758/0.3621/0.3714	Probably Damaging	Disease Causing	Deleterious
p.30229N>S	SUD (8), SUD/SIDS (3), ARVC, BrS, HCM (3)	16	yes	rs149001703	NA	0.8111/0.097/0.5757	Benign	Disease Causing	Neutral
p.31170R>C	LQT (2), SUD (3), CPVT, DCM (2)	8	yes	rs56273463	NA	0.9761/0.2302/0.7372	Probably Damaging	Disease Causing	Deleterious
p.31321V>I	LQT, SUD (2), HCM	4	yes	rs34924609	NA	0.6481/0.1623/0.4969	Benign	Disease Causing	Neutral
p.33028T>I	ARVC, DCM, SUD (2), HCM	5	yes	rs55842557/Likely benign	NA	0.6818/0.0793/0.4918	Benign	Polymorphism	Deleterious

BrS: Brugada Syndrome; LQT: Long QT Syndrome; SQT: Short QT Syndrome; CPVT: Catecholaminergic Polymorphic Ventricular Tachycardia; ARVC: Arrhythmogenic Right Ventricular Cardiomyopathy; HCM: Hypertrophic Cardiomyopathy; DCM: Dilated cardiomyopathy; LVNC: Left ventricular Non-Compaction; SUD: Sudden Unexplained Death; SIDS, Sudden Infant Death Syndrome; NA: Not Available. HGDM: Human Gene Mutation Database; MAF: Minor Allele Frequency; EA: European American; AA: African American; All: all populations. Numbers in the parentheses: Refer to number of variants repeated in the specific disease/syndrome.
